# Quantitative trait loci and differential gene expression analyses reveal the genetic basis for negatively associated β-carotene and starch content in hexaploid sweetpotato [*Ipomoea batatas* (L.) Lam.]

**DOI:** 10.1007/s00122-019-03437-7

**Published:** 2019-10-08

**Authors:** Dorcus C. Gemenet, Guilherme da Silva Pereira, Bert De Boeck, Joshua C. Wood, Marcelo Mollinari, Bode A. Olukolu, Federico Diaz, Veronica Mosquera, Reuben T. Ssali, Maria David, Mercy N. Kitavi, Gabriela Burgos, Thomas Zum Felde, Marc Ghislain, Edward Carey, Jolien Swanckaert, Lachlan J. M. Coin, Zhangjun Fei, John P. Hamilton, Benard Yada, G. Craig Yencho, Zhao-Bang Zeng, Robert O. M. Mwanga, Awais Khan, Wolfgang J. Gruneberg, C. Robin Buell

**Affiliations:** 1 International Potato Center, ILRI Campus, Old Naivasha Road, P.O. Box 25171-00603, Nairobi, Kenya; 2 North Carolina State University, Raleigh, NC 27695 USA; 3 International Potato Center, Av. La Molina 1895, Lima, Peru; 4 Michigan State University, East Lansing, MI 48824 USA; 5 International Potato Center, Kampala, Uganda; 6 International Potato Center, Kumasi, Ghana; 7 University of Queensland, St. Lucia, Brisbane, QLD 4072 Australia; 8 Boyce Thompson Institute, Cornell University, Ithaca, NY 14853 USA; 9 National Crops Resources Research Institute (NaCCRI), Namulonge, P.O. Box 7084, Kampala, Uganda; 10 Plant Pathology and Plant-Microbe Biology Section, Cornell University, Geneva, NY 14456 USA; 11 University of Tennessee, Knoxville, TN 37996 USA

## Abstract

**Key message:**

β-Carotene content in sweetpotato is associated with the *Orange* and *phytoene synthase* genes; due to physical linkage of *phytoene synthase* with *sucrose synthase*, β-carotene and starch content are negatively correlated.

**Abstract:**

In populations depending on sweetpotato for food security, starch is an important source of calories, while β-carotene is an important source of provitamin A. The negative association between the two traits contributes to the low nutritional quality of sweetpotato consumed, especially in sub-Saharan Africa. Using a biparental mapping population of 315 F_1_ progeny generated from a cross between an orange-fleshed and a non-orange-fleshed sweetpotato variety, we identified two major quantitative trait loci (QTL) on linkage group (LG) three (LG3) and twelve (LG12) affecting starch, β-carotene, and their correlated traits, dry matter and flesh color. Analysis of parental haplotypes indicated that these two regions acted pleiotropically to reduce starch content and increase β-carotene in genotypes carrying the orange-fleshed parental haplotype at the LG3 locus. *Phytoene synthase* and *sucrose synthase,* the rate-limiting and linked genes located within the QTL on LG3 involved in the carotenoid and starch biosynthesis, respectively, were differentially expressed in Beauregard versus Tanzania storage roots. The *Orange* gene, the molecular switch for chromoplast biogenesis, located within the QTL on LG12 while not differentially expressed was expressed in developing roots of the parental genotypes. We conclude that these two QTL regions act together in a *cis* and *trans* manner to inhibit starch biosynthesis in amyloplasts and enhance chromoplast biogenesis, carotenoid biosynthesis, and accumulation in orange-fleshed sweetpotato. Understanding the genetic basis of this negative association between starch and β-carotene will inform future sweetpotato breeding strategies targeting sweetpotato for food and nutritional security.

**Electronic supplementary material:**

The online version of this article (10.1007/s00122-019-03437-7) contains supplementary material, which is available to authorized users.

## Introduction

Sweetpotato [*Ipomoea batatas* (L.) Lam. (2*n* = 6*x* = 90)] has been successfully demonstrated to provide provitamin A biofortification in sub-Saharan Africa (SSA) due to its ability to accumulate high levels of β-carotene, especially in the orange-fleshed types (Mwanga et al. 2011; Low et al. 2009, 2017). However, the crop had been historically grown in SSA as a food security crop with consumers preferring predominantly high dry matter types which are white-fleshed or yellow-fleshed and less nutritious (Low et al. 2017). As a major constituent of dry matter, starch accounts for about 75% of human caloric intake (Naeem et al. 1997), and consequently, these adapted varieties have been naturally selected to have relatively high starch content. Additionally, such adapted varieties have specific textural characteristics after boiling that are preferred by local consumers. Texture and sweetness determine the eating quality of sweetpotato, both of which depend on the quality and quantity of carbohydrates such as cellulose, hemicellulose, pectin, starch, and sugars (Reeve 1967). Starch is the most important carbohydrate in sweetpotato storage root and its composition, size, and shape of granules contributes to eating quality (Reeve 1967; Kitahara et al. 2017).

β-carotene (BC) is the predominant carotenoid that accumulates in orange-fleshed sweetpotato storage roots and is a precursor of vitamin A. However, the understanding of the genetic basis of accumulation of carotenoids in underground storage roots is still fragmentary in many crops including sweetpotato (Carvalho et al. 2016). Carotenoid biosynthesis occurs in plastids of various forms. In seeds, roots, and tubers, carotenoid biosynthesis occurs in amyloplasts which are starch-storing plastids important for energy storage and gravitropism (Jarvis and Lopez-Juez 2013; Sun et al. 2018). Naturally, amyloplasts store carotenoids in small quantities typically in the form of lutein, zeaxanthin, and violaxanthin (Wurtzel et al. 2012). However, among roots and tubers, sweetpotato stands out for its capacity to induce modification of amyloplasts into crystalline-type carotenoid sequestration substructures called amylochromoplasts, which not only alter the carotenoid storage capacity, but also biosynthesis, causing higher accumulation of β-carotene in sweetpotato (Zhang et al. 2014). Unlike most staple crops where strategies are needed to manipulate carotenoid biosynthesis and degradation and/or plastid sink strength (Sun et al. 2018) to improve nutritional quality, these processes naturally occur in sweetpotato and can be readily selected in breeding populations.

The modification of amyloplasts, which are the energy stores within the plant cell to store carotenoids, results in starch biosynthesis and carotenoid biosynthesis competing for carbon that leads to the negative association reported in several crops including citrus (Cao et al. 2015), potato (Mortimer et al. 2016), and sweetpotato (Yada et al. 2017). Application of quantitative and population genetic principles, combined with improved experimental and statistical designs, has led to the release of many improved sweetpotato varieties, including orange-fleshed ones, in several countries within SSA recently (Gruneberg et al. 2015; Andrade et al. 2016). However, the actual adoption of many improved orange-fleshed varieties has been limited in part by the negative starch/β-carotene correlation and the yet undefined textural characteristics. Therefore, understanding the genetic architecture surrounding the negative association between starch and β-carotene and the possibility of breaking this linkage is an important objective of breeding programs targeting sweetpotato for food and nutritional security. Although most genes involved in carotenoid and starch/carbohydrate metabolic pathways are well characterized (Yuan et al. 2015) even in sweetpotato (Kang et al. 2017), insights into regulation of the pathways are still generally lacking. Additionally, these metabolic pathways have been studied independent of each other and no study has considered the molecular mechanisms driving the association between starch and carotenoids.

Hexaploid sweetpotato has two closely related sub-genomes (B_1_B_1_B_2_B_2_B_2_B_2_; Shiotani and Kawase 1989; Kriegner et al. 2003; Yang et al. 2017). Genetic analyses of sweetpotato are complicated by factors associated with polyploidy and heterozygosity. Such factors include the presence of multiple alleles at marker loci and differential allele dosage across homeologous chromosomes, the possibility of both bivalent and multivalent formation during meiosis, and the possibility of preferential pairing during meiosis (Dufresne et al. 2014). Compared to other crops, genomic tools that could be applied to expedite the breeding process in sweetpotato have been generally lacking due to the complexity of the genome and the relatively small critical mass of scientists working in this crop. However, due to the significant contribution of sweetpotato to humanity, more resources in the recent past have been deployed to develop genomic tools that address this complexity. In the current study, we demonstrate the robustness of reference genomes from two diploid relatives of sweetpotato, *Ipomoea trifida* and *Ipomoea triloba* (Wu et al. 2018), and new bioinformatic tools for polyploids (Wadl et al. 2018; Mollinari et al. 2019; Pereira et al. 2019) in aiding the understanding of genetic architecture of important traits in sweetpotato breeding for SSA. We quantified starch and β-carotene together with their correlated traits, dry matter (DM) and flesh color (FC), in a segregating population. We report on the quantitative trait loci (QTL) and differentially expressed genes within those loci and explore the negative starch/β-carotene association.

## Materials and methods

### Field experiments and laboratory analyses of traits

Data are reported from field experiments carried out in five environments in Peru. The first four environments were grown in Ica, a coastal desert town in the south of Peru, with two treatments (terminal drought and control experiments) for two seasons, while the fifth environment was located at San Ramon in the humid tropics at the beginning of the jungle, under optimal conditions. The terminal drought treatment was imposed by stopping irrigation at 70 days after transplanting (DAT) until harvest time at 120 DAT, while the control treatment was irrigated throughout the experiment. The environments were coded as follows: Ica16C, Ica16D, Ica17C, Ica17D, to indicated control and drought treatments at Ica in 2016 and 2017, respectively, while the fifth environment is coded as SR16 to indicate San Ramon in 2016. Details on locations and growing conditions are described in Online Resource 1, as well as in Pereira et al. (2019). We evaluated 315 segregating progeny derived from a cross between Beauregard (B) and Tanzania (T) varieties. Both parents differ in several traits. A representative sample of three storage roots was taken from each field plot and all replications per environment (environment = an experiment with the complete set of replicated genotypes with varying treatments or seasons or locations). Samples were transported to the nutritional laboratory at the International Potato Center (CIP) for analysis of quality-related traits. The samples were processed by peeling, slicing, and freeze-drying. We measured four correlated traits: DM, starch, BC, and FC_P (flesh color in Peru). DM was measured as a percentage of laboratory dried samples against the fresh weight of 100 g. Starch and BC contents were measured on the freeze-dried samples using near-infrared reflectance spectroscopy (NIRS) according to Tumwegamire et al. (2011). As an indirect measure for β-carotene, FC_P was scored based on color charts developed in-house for sweetpotato at CIP, on a scale of 1–9 (from white to dark orange). This scale is based on the sweetpotato descriptors jointly developed by CIP, Asian Vegetable Research and Development Center (AVRDC), and the International Board for Plant Genetic Resources (IBPGR) and normally used when flesh color is measured in addition to β-carotene content (CIP/AVRDC/IBPGR 1991). To examine the representativeness of the data reported here for our target breeding environments in sub-Saharan Africa, we also analyzed flesh color scores taken from the same population evaluated in six environments of Uganda (FC_U = flesh color in Uganda) in east Africa, i.e., three locations over 2 years (2016 and 2017). The scale used to measure flesh color in Uganda is based on the CIP Color Chart by Burgos et al. (2009) which uses flesh color to estimate levels of β-carotene on a scale of 1–30 (1 = low β-carotene and 30 = high β-carotene), without the actual measurement of β-carotene. The environments Nam16 and Nam17 were evaluated in Namulonge (0°31′17.99″N and 32°36′32.39″E) during 2016 and 2017, respectively, environments Ser16 and Ser17 were evaluated in Serere (1°29′59.99″N and 33°32′59.99″E) during 2016 and 2017, respectively, whereas environments Kac16 and Kac17 were evaluated in Kachwekano (1°15′0″S and 29°57′0″E) during 2016 and 2017, respectively. Online Resource 2 shows the flesh color of the parents and part of the segregating population.

## Data analyses

### Genetic correlation between environments and broad-sense phenotypic heritability

Variance components were estimated by restricted maximum likelihood (REML) using ASReml-R in a mixed model assuming heterogeneity of variances and genetic correlations across environments. The experiments were analyzed following the 80 × 4 alpha lattice design used in field trials. Specifically, the following mixed model was applied:

$$y_{ijkl} = \mu + e_{l} + r_{k\left( l \right)} + b_{{j\left( {kl} \right)}} + (\varvec{t}_{i} )_{l} + \varepsilon_{ijkl} ,$$

where *y*_*ijkl*_ is the phenotype of the *i*th treatment in the *j*th block within the *k*th replicate at the *l*th environment, *μ* is the overall mean, *e*_*l*_ is the random effect of the *l*th environment ($l = 1, \ldots , L$; $L = 5 {\text{ or }}6$ depending on the trait) with $e_{l} \sim {\mathcal{N}}\left( {0, \sigma_{e}^{2} } \right)$, $r_{k\left( l \right)}$ is the random effect of the *k*th replicate ($k = 1, \ldots , K$; $K = 2 {\text{ or }} 3$ depending on the environment) at the *l*th environment with $r_{k\left( l \right)} \sim {\mathcal{N}}\left( {0, \sigma_{r\left( l \right)}^{2} } \right)$, $b_{{j\left( {kl} \right)}}$ is the random effect of the *j*th block ($j = 1, \ldots , J$; $J = 80$) within the *k*th replicate at the *l*th environment with $b_{{j\left( {kl} \right)}} \sim {\mathcal{N}}\left( {0, \sigma_{b\left( l \right)}^{2} } \right)$, $\varvec{t}_{\varvec{i}} = \left( {t_{i1} , ..,t_{il} ,..,t_{iL} } \right)^{T}$ is the random effect of the *i*th treatment ($i = 1, \ldots , I$; $I = 318$) across the $L$ environments where $\varvec{t}_{\varvec{i}} \sim {\mathcal{N}}\left( {0,\sum } \right)$ with the variance covariance matrix $\sum$ expressing the genetic variances and covariances across the *L* environments, and *ɛ*_*ijkl*_ is the random residual error with $\varepsilon_{ijkl} \sim {\mathcal{N}}\left( {0, \sigma_{\left( l \right)}^{2} } \right)$. The ∑ matrix differed depending on the trait. For DM, starch, and FC_P, the variance–covariance matrix ∑ was unstructured. For BC, the variance–covariance matrix ∑ was obtained by a second-order factor analytic model (FA2), and for FC_U, the ∑ was obtained by a first-order factor analytic model (FA1). In addition to the genetic correlations between pairs of environments estimated above, general broad-sense heritabilities (*H*^2^) were approximated as the ratio between genotypic and phenotypic variances, averaged out across the different environments, from the estimated variance components given in the ASReml-R output. The raw data and joint adjusted means from this analysis used in QTL mapping are provided in Online Resource 3.

### Quantitative trait loci (QTL) analyses

The population was genotyped using GBSpoly, a genotype-by-sequencing method optimized for highly heterozygous and polyploid genomes (Wadl et al. 2018). In summary, the concentration of DNA samples was normalized after quality check on 1% agarose gel and quantification based on the PicoGreen fluorescence-based assay. Next-generation sequencing 64-plex libraries were made based on the GBSpoly protocol (Wadl et al. 2018), while 125 bp single-end sequencing was performed on a total of 40 sequencing lanes (8 lanes for each of the 5 libraries) of the Illumina HiSeq 2500 platform. We aligned the GBS tags against the diploid relatives of sweetpotato, *I. trifida* and *I. triloba* reference genomes (Wu et al. 2018) using Bowtie2 (Langmead and Salzberg 2012), and obtained the allele read counts per locus using the Tassel-GBS pipeline (Glaubitz et al. 2014) modified for polyploids (Pereira et al. 2018). The read counts were ultimately used for dosage calling in the software SuperMASSA (Serang et al. 2012) with the help of VCF2SM script (Pereira et al. 2018). Single-nucleotide polymorphisms (SNPs) with read depth < 20, > 25% missing data and significant segregation distortion (*p* < 5 × 10^−4^) were filtered out (Mollinari et al. 2019). A genetic linkage map was developed using MAPpoly software for linkage mapping in polyploids (Mollinari and Garcia 2019) based on the dosage calls. The integrated map had 15 linkage groups (LGs) containing 30,684 GBSpoly-generated and phased SNPs with a total length of 2708.4 cM and an average distance between markers of about 0.09 cM (Mollinari et al. 2019). The phasing procedure was based on parental genotypes, and their inheritance patterns were observed in the offspring individuals. More specifically, the phasing algorithm used the LOD scores of pairwise markers analysis as a primary source of information to sequentially position the allelic variants in specific homologs. For situations where pairwise analysis had limited power to detect the linkage phase (LOD score < 10.0), the algorithm used the likelihood of multiple markers in a hidden Markov model. Further details are described in Mollinari et al. (2019). The QTL genotype conditional probabilities based on the map and the adjusted genotypic means above were then used in QTL mapping using the QTLpoly software (https://github.com/guilherme-pereira/qtlpoly), a QTL mapping approach for polyploids (Pereira et al. 2019).

In brief, we used a random-effect multiple interval mapping (REMIM) model where score-based tests (Qu et al. 2013) were performed every 1 cM following a stepwise method. First, the forward search added one QTL at a time into a multiple QTL model using a less conservative threshold (*p* value < 0.01). Then, the backward elimination tested each QTL again conditional to all the others in the model using a more conservative threshold (*p* value < 0.001). Under the more conservative threshold, forward and backward procedures were repeated until no more QTLs were added or dropped from the model. We avoided a region of 15 cM on either side of QTLs already in the model when searching for a new QTL. QTL heritabilities ($h_{\text{QTL}}^{2}$) were computed as the ratio of QTL and total variances, which were estimated using restricted maximum likelihood (REML) as implemented in the R package Sommer (Covarrubias-Pazaran 2016).

### Gene expression profiling

Gene expression profiling of root development in Beauregard was reported previously by Wu et al. (2018) and included replicated (four biological replicates) RNA-sequencing datasets for total roots at 10 days after transplanting (DAT) and 20 DAT, fibrous roots at 30 DAT, 40 DAT, and 50 DAT, and storage roots at 30 DAT, 40 DAT, and 50 DAT. Tanzania plants were grown in parallel with the “Beauregard” plants as previously described (Wu et al. 2018) and harvested at 10, 20, 30, 40, and 50 DAT; four replicates were generated. The roots were classified at 30, 40, and 50 DAT into fibrous and storage roots based on diameter as described by Wu et al. (2018). Tanzania RNA was isolated, and libraries were constructed and sequenced as described by Wu et al. (2018). To assess expression abundances, both Beauregard and Tanzania RNA-sequencing reads were cleaned, aligned to the *I. trifida* genome, and fragments per kb exon model per million mapped reads (FPKM) were determined as previously described in Lau et al. (2018). For the final FPKM matrix, genes encoded by the chloroplast were removed. Pearson’s correlation coefficients and principal component analyses were performed in R (v3.5.0) using the log2 (FPKM + 1) values. To identify differentially expressed genes (DEG), uniquely mapping reads overlapping gene models were counted using HTSeq (v0.6.1p1; Anders et al. 2015) with the following options: -stranded = reverse -minaqual = 10 -type = exon -mode = union; DESeq2 (v1.22.2; Love et al. 2014) was used for detecting differential expression. Differential expression between Beauregard and Tanzania for each timepoint and root type combination was conducted using the contrasts function in DESeq2 to test whether log2 fold change was equal to 0 for each pair of contrasts. A log2 fold-change (lfc) threshold of 2, along with an adjusted *p* value cutoff of 0.01 was used. The DESeq2 function “lfcshrink” was used to help restrain the high log2 fold changes of genes that had low expression values. Gene ontology (GO) term enrichment tests were performed on sets of DEGs using the “weight01” algorithm and Fisher’s exact test implemented in the R package topGO (v2.34.0; Alexa and Rahnenfuhrer 2019) using all genes with GO terms as background. The “p.adjust” function in R was used to implement the FDR method (Benjamini and Hochberg 1995) and correct the *p* values for multiple testing. The *p* values were then filtered at a level of 0.05.

### Locating the markers of QTL within the reference genome to infer candidate genes

We used the markers at the peak of QTL identified for each trait and their support intervals to query the reference genome using V3 of the *I. trifida* genome assembly (Wu et al. 2018). An initial set of candidate carotenoid biosynthetic and metabolism genes were obtained from Wu et al. (2018) and refined using alignments against UniRef100.

## Results

### Phenotypic performance: Impact of environment on traits

**Fig. 1:**
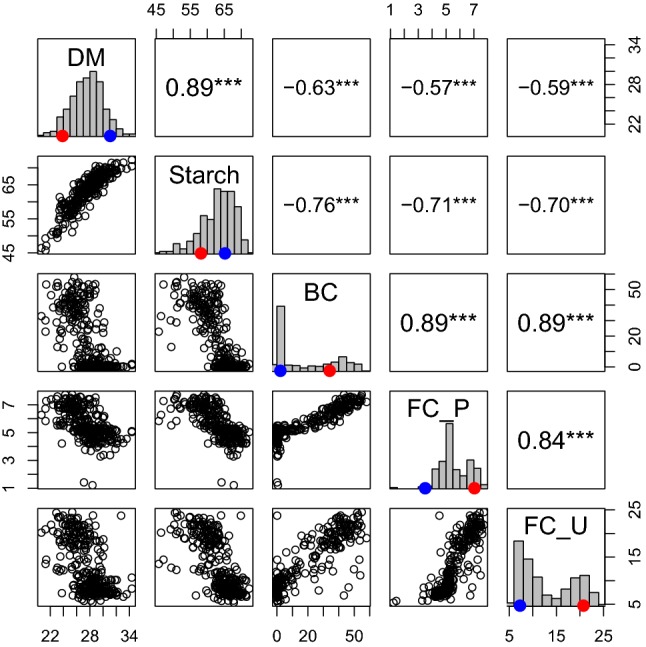
Correlation coefficients (***p < 0.001) among genotypic means for dry matter (DM), starch, β-carotene (BC), flesh color in Peru (FC_P) and Uganda (FC_U). The red and blue dots indicate the mean value of Beauregard and Tanzania, respectively

We measured dry matter, starch, β-carotene, and flesh color of a biparental population segregating for these traits and grown in multiple environments in Peru and Uganda (Online Resource 3). Genetic correlations between each pair of environments were high for the four traits measured (ranging from *r* = 0.70 to *r* = 0.99) including genetic correlations between environments in Peru and Uganda for FC (Online Resource 4). Consequently, broad-sense heritability (*H*^2^) estimates were also high for all traits: DM = 0.61, starch = 0.77, BC = 0.91, FC_P = 0.92, and FC_U = 0.89 (Online Resource 4). Based on the predicted means, DM was highly positively correlated with starch (*r* = 0.89) and negatively correlated with BC (*r* = − 0.63), FC_P (*r* = − 0.57) and FC_U (*r* = − 0.59). BC was negatively correlated with starch (*r* = − 0.76) and positively with FC_P (*r* = 0.89) and FC_U (*r* = 0.89). As expected from the genetic correlation above, FC_P and FC_U were highly and positively correlated (*r* = 0.84), but negatively correlated with starch (*r* = − 0.71 for FC_P and *r* = − 0.70 for FC_U, respectively; Fig. 1).

### Quantitative trait loci (QTL) associated with quality-related traits

**Fig. 2:**
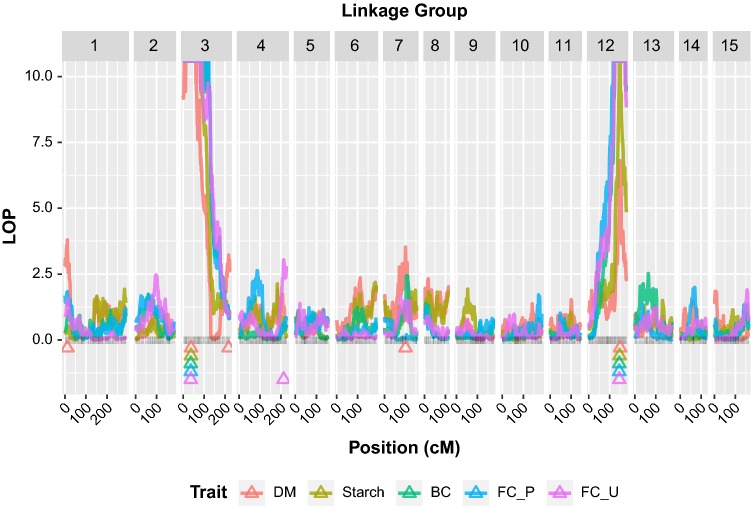
Quantitative trait loci (QTL) profiles as LOP = −log10(*p*) for dry matter (DM), starch, β-carotene (BC), flesh color in Peru (FC_P) and Uganda (FC_U) along the Beauregard × Tanzania sweetpotato genetic map. Triangles show the location of QTL peaks

We analyzed QTL for four correlated traits in sweetpotato: DM, starch, BC, and FC (FC_P and FC_U) based on an integrated genetic map of the 15 LGs of sweetpotato spanning 2,708.4 centimorgans (cM). This was made possible with the availability of anchored reference genomes from two diploid relatives of sweetpotato, *I. trifida* and *I. triloba* (Wu et al. 2018), and new linkage and QTL mapping methods for polyploids (Mollinari et al. 2019; Pereira et al. 2019). Except for DM and FC_U, which had additional minor QTL on LG1, LG3 and LG7 (DM), and LG4 (FC_U), the major QTL explaining the observed variation for the traits in the mapping population were co-localized on LG3 and LG12 (Fig. 2, Online Resource 5). The co-localized QTL on LG3 with peaks for all traits between 36.14 and 37.44 cM explained 37.4%, 51.2%, 50.2%, 53.6%, and 48.8% of observed variation in DM, starch, BC, FC_P, and FC_U, respectively (Table 1). The co-localized QTL on LG12 with peaks for all traits between 146.02 and 150.05 cM explained 11.2%, 17.0%, 29.0%, 29.3%, and 27.7% of observed variation in DM, starch, BC, FC_P, and FC_U, respectively. The additional QTL for DM on LG1, LG3, and LG7 explained 6.9%, 6.2%, and 6.0%, respectively, while the additional QTL on LG4 for FC_U explained 3.2% of the observed variation. The physical positions of the QTL peaks as well as their support intervals are shown in Online Resource 6.

**Table 1:** Summary of quantitative trait loci (QTL) for dry matter (DM), starch, β-carotene (BC), flesh color in Peru (FC_P) and flesh color in Uganda (FC_U)

Trait	QTL	LG^a^	Pos^b^ (cM)	SI^c^ (cM)	Score	*p* value	$\sigma_{\text{QTL}}^{2}$	$h_{\text{QTL}}^{2}$^d^
DM	1	1	11.41	0.00–21.02	158.93	1.59e−04	0.6590	0.069
	2	3	37.44	4.68–62.06	604.15	< 1.00e−16	3.5713	0.374
	3	3	217.13	193.17–222.06	133.93	6.00e−04	0.5904	0.062
	4	7	100.26	65.46–113.08	151.34	2.99e−04	0.5702	0.060
	5	12	150.05	146.02–155.04	264.05	1.57e−07	1.0657	0.112
Starch	1	3	37.44	0.00–84.32	849.10	< 1.00e−16	21.3694	0.512
	2	12	147.31	146.02–150.05	435.34	< 1.00e−16	7.0820	0.170
BC	1	3	36.14	0.00–121	1067.07	< 1.00e−16	336.9740	0.502
	2	12	146.02	124.12–176.02	828.83	< 1.00e−16	194.5965	0.290
FC_P	1	3	36.14	0.00–122.1	1262.29	< 1.00e−16	1.1444	0.536
	2	12	146.02	117.02–180.05	944.49	< 1.00e−16	0.6259	0.293
FC_U	1	3	36.14	0.00–88.10	1064.73	< 1.00e−16	28.5757	0.488
	2	4	213.05	200.09–227.13	135.98	9.20e−04	1.8504	0.032
	3	12	146.02	121.41–176.02	794.68	< 1.00e−16	16.2076	0.277

^a^Linkage group

^b^Position in centiMorgans

^c^Support interval in centiMorgans

^d^Proportion of total variance explained by a QTL

**Fig. 3:**
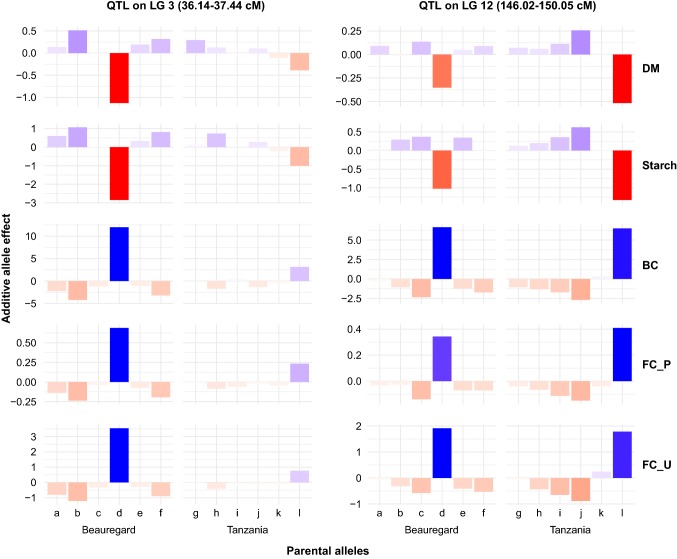
Additive allele effect estimates for co-localized QTL on LG3 and LG12 (map position of the QTL peaks in parenthesis) for dry matter (DM), starch, β-carotene (BC), and flesh color in Peru (FC_P) and Uganda (FC_U). Letters *a* through *f* and *g* through *l* represent the six haplotypes for the specific QTL for Beauregard and Tanzania, respectively

Based on the QTL results, we examined the contribution of parental haplotypes in the major QTL on LG3 and LG12 (Fig. 3). We observed that both parents contributed major alleles with similar allelic effects on the traits at the LG12 QTL, but only Beauregard contributed the major allelic effect with effect on traits at the LG3 QTL (Fig. 3). In fact, additive allele effect estimates from these traits show the same contributing haplotypes in completely opposite directions, i.e., the alleles (*d* from QTL on LG3, and *d* and *l* from QTL on LG12) that were involved in decreasing the means for DM and starch were the same alleles responsible for increasing the means for BC and FC (Fig. 3). These results indicate that haplotypic variations within these two regions are responsible for the observed negative association between starch and BC in sweetpotato. Given that the two parents are contrasting for these traits, these results indicate that even though both contribute haplotypes with similar allelic effects within the QTL on LG12, the interaction between these alleles with those within the QTL on LG3 determines the accumulation or lack of accumulation of β-carotene in the storage roots.

### Differential gene expression during storage root development

The co-localization of QTL for the above traits indicated that starch (a major constituent of DM) and carotenoid biosynthesis and accumulation were connected through the interaction of alleles within QTL on LG3 and LG12. To identify candidate genes within our QTL, gene expression profiling datasets from developing roots of Beauregard and Tanzania were examined. Replicated global gene expression profiles were generated from whole Beauregard and Tanzania roots at 10 DAT and 20 DAT as well as fibrous and storage roots at 30, 40, and 50 DAT (Online Resource 7, Online Resource 8). Pearson’s correlation analyses of replicates revealed a high degree of reproducibility between biological replicates (Online Resource 9). Principal component analyses (PCA) showed separate clustering of the 30 DAT, 40 DAT, and 50 DAT fibrous roots in Beauregard compared to their corresponding storage roots suggestive of programmed changes in expression profiles in fibrous versus storage roots (Online Resource 10). However, for Tanzania, only temporal separation of the samples was observed and no differentiation of fibrous roots relative to the storage root samples was observed in the PCA plot. Thus, while Tanzania roots were sampled for fibrous versus storage roots based on root diameter (≤ 2.5 mm and ≥ 2.5 mm, respectively), they were not well differentiated based on gene expression profiles, consistent with their longer maturity period (150 days) relative to Beauregard (90 days). Indeed, determination of differentially expressed genes with DESeq2 of storage roots versus fibrous roots for Beauregard and Tanzania revealed substantially more differentially expressed genes in Beauregard relative to Tanzania at 30, 40, and 50 DAT (Online Resource 11) with no differentially expressed genes in Tanzania at 40 DAT storage roots versus fibrous roots. Gene ontology (GO) enrichment analysis of differentially regulated genes in Beauregard at 30 DAT revealed up-regulation of genes involved in transcription regulation (Online Resource 12), while at 40 DAT, multiple GO terms involved in cell wall modification were observed in up-regulated genes, and at 50 DAT, GO terms associated with starch biosynthesis, response to sucrose, cell wall organization, and regulation of meristem growth were observed in up-regulated genes. In Tanzania, similar GO term enrichment was observed (Online Resource 12), but the numbers of genes were substantially less than and were not temporally synchronized with Beauregard.

### Candidate genes within QTL of interest

**Fig. 4:**
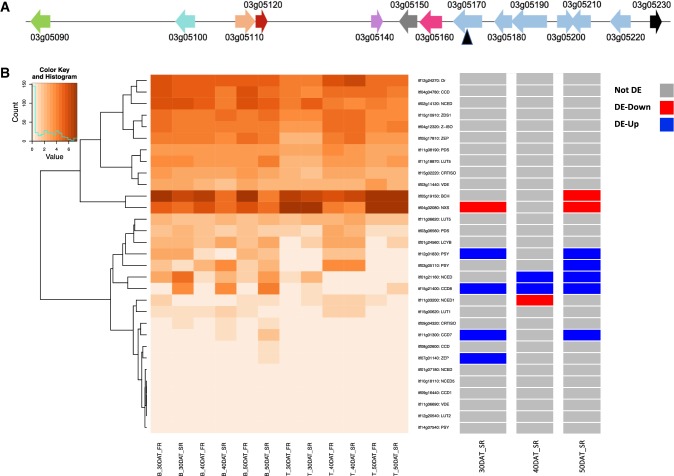
Characterization of the QTL on LG3 underlying starch and β-carotene. **a** Genes are noted by arrows: Homeodomain-like superfamily protein (green); sucrose synthase (aqua), phytoene synthase (lt. orange), glutathione S-transferase (burgundy), AMP-dependent synthetase and ligase family protein (lilac), RAB homolog (gray), polyamine oxidase (magenta), P-loop containing nucleoside triphosphate hydrolase superfamily protein (Lt. blue), and conserved hypothetical (black). Black arrowhead denotes marker S3_3185578. **b** Left panel: Expression abundances (log2 fragments per kilobase per exon model per million mapped reads (FPKM)) of candidate genes involved in carotenoid metabolism are shown in the heat map below each gene for Beauregard (B) and Tanzania (T) for storage roots (SR) and fibrous roots (RF) at 30, 40, and 50 days after transplanting (DAT). Key code value indicates log2 FPKM and count indicates the number of samples (sample = one gene per sampling) with that FPKM value shown as a histogram. Gene identifiers and gene name abbreviations are listed to the right of the heat map. BCH, β-carotene hydrolase; CCD, carotenoid cleavage dioxygenases; CRTISO, carotene isomerase; LUT, lutein deficient; LYCB, lycopene b-cyclase; NCED, 9-cis-epoxycarotenoid dioxygenase; NXS, neoxanthin synthase; OR, ORANGE protein; PDS, phytoene desaturase; PSY, phytoene synthase; VDE, violaxanthin de-epoxidase; ZEP, zeaxanthin epoxidase; ZDS, zeta-carotene desaturase; Z-ISO, z-carotene isomerase. Right panel: Differentially expressed genes based on the comparison of Beauregard versus Tanzania storage roots (color figure online)

Starch and carotenoid metabolisms are well characterized in plants, and the major pathways are well documented. The colocated QTL covered a large area that contained many genes (Online Resource 5, Online Resource 7, Online Resource 8) as our mapping population (315 progenies) was not large enough to narrow down the QTL region to single, causative genes. Therefore, we focused on the major reported rate-limiting genes of starch and carotenoid metabolism within these regions including genes differentially expressed between Beauregard and Tanzania which differ significantly in their carotenoid content. Wu et al. (2018) reported a significant single-nucleotide polymorphism (SNP) located on LG3 within the *phytoene synthase* gene (*PSY*), a rate-limiting gene in the carotenoid biosynthesis and degradation pathway (Sun et al. 2018) associated with flesh color in 16 orange-fleshed and white-fleshed parents of an African breeding population. The peak of the QTL on LG3 shared by BC and FC is at 2,994,719 bp, and the peak shared by DM and starch is at 3,185,578 bp. The *PSY* gene (*itf03g05110*) is located between 3,117,946 and 3,122,156 bp and in close proximity to these two peaks (Fig. 4a). Gene expression comparisons between Beauregard and Tanzania are confounded by the longer maturity of Tanzania relative to Beauregard. However, Beauregard accumulates carotenoids in both fibrous and storage roots starting at 30 DAT (Gemenet, pers. comm.), while Tanzania fails to accumulate significant carotenoids even at maturity (Online Resource 2). Thus, to determine if the BC QTL on LG3 was associated with expression differences of *PSY*, we examined gene expression profiles in 30, 40, and 50 DAT fibrous and storage roots of Beauregard and Tanzania. *PSY* was expressed in all sampled Beauregard roots with peak levels present in 40 DAT storage roots (Fig. 4b, Online Resource 13). *PSY* was also expressed in Tanzania roots with the highest expression abundances in 10 DAT, 20 DAT, and 40 DAT fibrous and storage roots with no expression in 30 DAT storage roots and extremely low expression observed at 50 DAT fibrous and storage roots. In 50 DAT storage roots, *PSY* was differentially expressed between Beauregard and Tanzania (log2fc = 2.34, adj. *p* value 4.2E−6, respectively; Fig. 4b, Online Resource 13).

Three other genes involved in carotenoid biosynthesis and metabolism underlie carotenoid-related QTL including *15*-*cis*-*phytoene desaturase* (itf03g06560) and *violaxanthin de*-*epoxidase* (itf03g11440) that underlie QTL for BC and FC and *lycopene β*-*cyclase* (itf04g32080) which underlies FC (Fig. 4b, Online Resource 13). Of specific interest is itf04g32080 as similarly to *PSY*, it was associated with flesh color in a panel of 16 orange-fleshed and white-fleshed parents of an African breeding population (Wu et al. 2018); itf04g32080 is down-regulated between Beauregard and Tanzania in 30 and 50 DAT storage roots (Fig. 4b, Online Resource 13).

Interestingly, *PSY* is within 12.2 kb of *sucrose synthase* (*SuSY; itf03g05100*), with no intervening genes (Fig. 4a). There is increasing evidence that starch biosynthesis in amyloplasts is dependent on ADP-glucose synthesized in the cytosol via *SuSY* which is transported to the amyloplasts. *SuSY* was expressed at similar levels in 10 and 20 DAT roots of both Beauregard and Tanzania (Online Resource 7, Online Resource 8); however, beginning at 30 DAT in Beauregard, fibrous and storage roots differ in *SuSY* expression abundance with no expression detected in Beauregard storage roots at 50 DAT. This contrasts with Tanzania in which *SuSY* levels remain elevated throughout the time course in both fibrous and storage roots. In 30 DAT storage roots, *SuSY* was differentially regulated in Beauregard relative to Tanzania (log2fc = − 2.59; adj. *p* value = 1.8E−12; Online Resource 14).

**Fig. 5:**
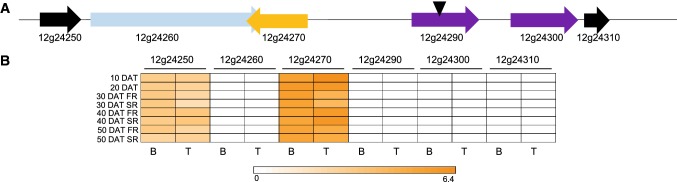
Region on LG12 underlying β-carotene levels. **a** Genes are noted by arrows: conserved hypothetical (black), P-loop containing nucleoside triphosphate hydrolase superfamily protein (lt. blue), Orange (orange), and SU(VAR)3-9 homolog (purple). Black arrowhead denotes marker S12_ 22131994. **b** Expression abundances (log2 fragments per kilobase per exon model per million mapped reads) are shown in the heat map below each gene for Beauregard (B) and Tanzania (T). *DAT*, days after transplanting, *SR* storage roots, *FR* fibrous roots (color figure online)

The peak of the QTL associated with BC on LG12 was located within *itf12g24290* (marker S12_ 22131994; Fig. 5a) which is annotated as SU(VAR)3-9. Nearby (5.7 kb) is a homolog of the *ORANGE (Or)* gene (*itf12g24270*), originally identified in cauliflower, and responsible for acting on and regulating *PSY* to allow modification of amyloplasts into chromoplasts (Lu et al. 2006). *Or*^2018^ is hypothesized to be the molecular switch for chromoplast biogenesis (Sun et al. 2018). A homolog of the *Or* gene (*IbOr*) was cloned from sweetpotato and shown to function in the accumulation of carotenoids in sweetpotato storage roots (Kim et al. 2013). Interestingly, *itf12g24270* but not *itf12g24290* is highly expressed in both Beauregard and Tanzania sweetpotato roots throughout our time course, indicating that *Or* is the major gene associated with carotenoid/starch accumulation at this major QTL locus (Fig. 5b); *Or* was not differentially expressed in any comparison of Beauregard versus Tanzania roots using our log2fc cutoff of 2. Another gene of interest within this QTL region is *itf12g26180*, a *Glycogen/starch synthase, ADP*-*glucose type*, which synthesizes starch from the *SuSY*-catalyzed cytosolic ADP-glucose precursor.

## Discussion

We have applied novel methods to map and study QTL in hexaploid sweetpotato. Compared to previous mapping efforts in sweetpotato where mapping was based on independent homeologous groups, it is now possible to use SNP dosage markers to recover all the haplotypic information and to build a completely integrated genetic map (Mollinari et al. 2019). We were able to distinguish 12 different haplotypes corresponding to the six possible alleles at a locus by two hexaploid parents with a potential of up to 400 possible allele combinations at a given locus in progeny. Conditional probabilities of the (400) QTL genotypes at each locus were estimated based on the grouped, ordered, and phased map based on hidden Markov models adapted for polyploids. Using this method, multiple QTL mapping is performed, so that the search for new QTL is conditioned to all the other QTL already in the model (Pereira et al. 2019). Although previous studies in sweetpotato have reported QTL for quality-related traits including β-carotene (flesh color), dry matter, and starch (Cervantes-Flores et al. 2011; Zhao et al. 2013; Xiao-xia et al. 2014; Zhang et al. 2016; Yada et al. 2017), such studies following the pseudo-testcross approach have not contributed substantially towards applied breeding as they present a challenge in comparison with other studies. The current study provides a major improvement in the potential of marker-assisted breeding in sweetpotato.

We present results showing relatively high genetic correlations among pairs of environments and broad-sense heritability across environments, indicating that the studied traits are less affected by the environment and therefore minimal genotype-by-environment interaction is expected. These findings imply that the results presented here are likely to be repeatable across other environments. For most traits, several progenies had mean values higher than the better parent. These can be attributed to segregation and reassortment of alleles due to hybridization or to transgressive inheritance (Goulet et al. 2017). Evidence of transgressive inheritance is provided in the case of BC, for instance, where both parents contribute similar additive allele haplotypes on LG12 that increase BC and reduce starch, yet the parents are contrasting for the two traits. This negative association between β-carotene and starch content is well established in sweetpotato (Gruneberg et al. 2005; Cervantes-Flores et al. 2011; Yada et al. 2017) and citrus (Cao et al. 2015), among other crops.

The *PSY* locus, which is a rate-limiting gene in carotenoid biosynthesis, was previously reported to be associated with flesh color in 16 parents of an east African sweetpotato breeding population in Uganda (Wu et al. 2018), through a candidate gene association mapping approach. According to Foss et al. (2007), a phenotype is affected by both cis-acting (local) variation on the actual genes affecting the trait, as well as transacting (distant) variation, e.g., transcription factors, which may be located in a distant region of the genome away from the target gene. We were able to show cis- and trans-acting genes within QTL on LG3 and LG12 explaining the negative association between starch and β-carotene content in sweetpotato storage roots. The finding that major genes involved in chromoplast biogenesis, starch biosynthesis, and rate-limiting genes for carotenoid biosynthesis were located within the two QTL regions, indicated that activity surrounding chromoplast biogenesis determines the level of carotenoids accumulated and displacement of starch. Our results agree with the findings of Lu et al. (2017) in sweet orange, showing that crystalline chromoplast development explained most of the accumulated carotenoids more than the actual carotenoid metabolic pathways genes. The *IbOr* gene (Goo et al. 2015) which is located within our QTL on LG12 interacts with *PSY* gene on LG3 to enhance chromoplast biogenesis and carotenoid accumulation (Lu et al. 2006). The *Or* gene is known for its post-transcriptional regulation of *PSY* protein level and enzyme activity (Sun et al. 2018; Ellison et al. 2018). Evidence of post-transcriptional activity of *Or* on *PSY* was shown by Zhou et al. (2015) where overexpression of *Or* did not have an effect on the expression of *PSY* in *Arabidopsis thaliana*, both in roots and leaves, rather mediated *PSY* protein levels and carotenoid content, in cases with high *PSY* expression. This is supported by our current results that show no differential expression of *IbOr* between Tanzania and Beauregard even though they differ in the amounts of carotenoids, suggesting that the identified QTL are not expression-based and that sequence differences in the loci may confer different phenotypes. For example, Tzuri et al. (2015) and Ellison et al. (2018) showed that a single “golden SNP” within the *Or* gene is responsible for carotenoid accumulation in melon and carrot, respectively. Candidate gene sequence analysis of the *IbOr* is still necessary to confirm if this is also the case for sweetpotato. Our results show that, although Tanzania is white-fleshed, it contains a haplotype at the *IbOr* locus that contributes additively to increase β-carotene content in individuals with a favorable *PSY* haplotype, such that transgressive segregation for β-carotene could be explained by a complementary gene action (Goulet et al. 2017) between both parents. The similar additive allelic effects contributed by both parents at the LG12 QTL locus agree with gene expression data for the *IbOr* gene where similar and high expression was observed for both Beauregard and Tanzania throughout the time course. The current results also suggest that the variation between accumulation of starch or β-carotene is determined by the haplotype variation present at LG3 as regulated by the LG12 locus. Given that at the time of sampling for gene expression profiling Tanzania had not started differentiation into clear storage roots, future analyses of differentially expressed genes using later development stages of storage roots could further confirm the *PSY* and *Or* relationship in orange-fleshed and non-orange-fleshed sweetpotato. Additionally, since both Beauregard and Tanzania express *PSY* and *IbOr*, expression of each gene individually does not confer the ability to accumulate carotenoids in storage roots. The activities of *PSY* and *Or* are important for our study because it is a rate-limiting step, but also relates to the biosynthesis of carotenoids in general. However, the level of β-carotene present in storage roots is a function of both biosynthetic and degradation processes. This is evidenced in our results by the identification of other carotenoid biosynthesis and degradation genes within the QTL. For instance, the noted *lycopene β*-*cyclase* (*itf04g32080*) converts lycopene into β-carotene, while *violaxanthin de*-*epoxidase* catalyzes the conversion of violaxanthin into antheraxanthin, both of which are downstream products of β-carotene degradation (Wu et al. 2018).

We hypothesize that the *SuSY* locus linked to *PSY* is involved in the starch–carotenoid balance in the chromoplasts. There is increasing evidence that ADP-glucose synthesized in the cytosol by *SuSY* is critical in determining the rate of starch synthesis in amyloplasts and other plastids and that sucrose and starch biosynthesis is directly connected by *SuSY* (Naeem et al. 1997; Baroja-Fernández et al. 2004; Muñoz et al. 2005). The current results are supported by our differential gene expression results for *SuSY*. A recent genome-wide association mapping study in cassava looking at both flesh color and dry matter showed the close association between *PSY* with flesh color and *SuSY* with DM, allowing them to hypothesize that the association between the two traits may be through physical linkage rather than pleiotropy (Rabbi et al. 2017). This would suggest that haplotype variation within *PSY* enhances regulation by the *IbOr* gene to allow chromoplast biogenesis and carotenoid accumulation and this process inhibits starch synthesis by inhibiting *SuSY* activity due to the close linkage between *SuSy* and *PSY*. Starch is composed of amylose and amylopectin, and several genes are known to be responsible for its biosynthesis, with the metabolic pathways for starch biosynthesis being different between source and sink (Ohdan et al. 2005). For starch biosynthesis in sink tissue, the main substrate is sucrose, which can be metabolized by *SuSY* to either uridine diphosphate glucose (UDP-glucose) or ADP-glucose (Stein and Ganot 2019). Dejardin et al. (1997) showed that *SuSY* was more important in explaining the rate of starch synthesis when compared to both *ADP*-*Glucose pyrophosphorylase* and *starch synthase* in pea embryos. Additionally, enhanced activity of *SuSY* increased starch content and ADP-glucose levels in maize endosperms (Li et al. 2013). Cytosolic ADP-glucose-supported starch synthesis was previously reported in amyloplasts of potato (Naeem et al. 1997) and wheat endosperm (Tetlow et al. 1994) with suggested species-specific differences on the relative importance of this pathway compared to that of glucose-1-phosphate + ATP, depending on the adenylate translocator present.

All of these studies demonstrate *SuSY* to be a major determinant of starch content in plant heterotrophic (non-photosynthetic) tissues. They therefore support our hypothesis that starch synthesis in sweetpotato storage roots is dependent on the ADP-glucose synthesized in the cytosol by *SuSY* and transported to the amyloplasts and that *SuSY* activity is inhibited during chromoplast biogenesis resulting in carotenoid synthesis and accumulation. Additionally, our results show a bimodal distribution for BC and a normal distribution for DM and starch. It is known that more than two genes affect starch biosynthesis, which is also a major constituent of DM (Ohdan et al. 2005). This is confirmed by our results where additional, minor effect QTL were found for DM. Furthermore, the QTL on LG3 and LG12 together explain a lesser observed variation for DM and starch when compared with the observed variation explained by the same QTL for BC and FC. This means therefore that there must be more small effect QTL that may be undetected. In addition, broad-sense heritabilities were also lower for DM and starch when compared to BC, FC_P, and FC_U, further corroborating the quantitative nature of DM and starch. Despite this, the fact that QTL at LG3 and LG12 jointly explain 48.6% and 68.2% of the total observed variation for DM and starch, respectively, supports our hypothesis that these two regions are the most important for both starch and carotenoid biosyntheses and inform the dynamics that surround their accumulation or displacement of each other.

Most studies examining starch and carotenoid biosynthesis and accumulation have been performed with single traits. To the best of our knowledge, this is the first study where a physical linkage between *SuSy* and *PSY* has been shown in addition to their association with the chromoplast biogenesis gene *Or*. Studies on the active mechanisms and regulation among the three candidate genes to further explain the negative association between starch and β-carotene are necessary. Future fine mapping within the current QTL regions offers an opportunity to study these putative candidate genes better. Toward their application in plant breeding, the analysis of haplotype variation within well-characterized candidate genes will enhance mining of desirable allelic haplotypes from natural populations or target breeding populations which can be selected using functional markers. For those pathways that are not very well defined yet, candidate genes will need to be characterized by the use of reverse genetics using transformation, RNA interference (RNAi), and mutant characterization. These will then inform breeding programs on the possibility of breaking genetic linkage among loci through recombination and selection of desirable haplotypes or to apply new crop improvement methods such as gene editing.

## Conclusion

Using novel methods, we are able to explore the genetic architecture surrounding the negative association between starch and β-carotene content in sweetpotato. Based on data presented here and reports in the literature, we suggest that *IbOr* gene on LG12 and *PSY* on LG3 act pleiotropically to modify amyloplasts into chromoplasts for synthesis and accumulation of β-carotene in orange-fleshed sweetpotato, and that this interaction is more important than the individual expression of each gene individually. We also suggest that this amyloplast modification affects starch content through the direct linkage between *PSY* and *SuSY* by inhibiting the activity of *SuSY* in storage roots of orange-fleshed sweetpotato, thereby reducing the ADP-glucose precursor available from the cytosol for starch synthesis in the amyloplasts. These results will be helpful in designing methods toward genomic-assisted breeding approaches in sweetpotato breeding programs, especially through haplotype variation analysis within the reported candidate genes. We also note that further research is still necessary to better understand the mechanisms and regulations underlying the negative association between starch and carotenoid content.

## Author contribution statement

AK, WJG, GCY, ZBZ, CRB, ZF, and LJMC designed and managed different aspects of the work; DCG, EC, ROM, AK, and WJG designed the experiments at CIP; DCG, FD, VM, AK, RTS, JS, ROM, and WJG carried out field experiments in Peru and Uganda; BAO, DCG, MNK, MD, MG, and GCY prepared the DNA/RNA samples and genotyped the mapping population; GB, TZF, DCG, and VM carried out the laboratory analysis of the traits; GDSP, MM, and ZBZ developed the genetic and QTL mapping software; ZF, CRB, JPH, and LJMC sequenced, annotated, and anchored the reference genome; ZF and CRB sequenced the RNA for gene expression profiling; DCG, GDSP, BDB, JCW, CRB, VM, and MM carried out the data analysis; DCG wrote the manuscript; and all authors read and approved the manuscript.

## Electronic supplementary material

Below is the link to the electronic supplementary material.

Online Resource 1: Growing conditions for sweetpotato mapping population (BT) experiments in five environments of Peru indicating, geographic positions, elevation, mean atmospheric temperature (μTemp), mean photosynthetically active radiation (μPAR), rainfall, relative humidity, soil conditions, planting designs, planting and harvesting dates (PDF 113 kb)

Online Resource 2: Flesh color segregation in progeny as compared to parents. The progeny shown are randomly selected from the 315 population (PNG 574 kb)

Online Resource 3: Raw data and joint adjusted means of the Beauregard x Tanzania mapping population used in QTL mapping for dry matter (DM), starch, β-carotene (BC), flesh color in Peru (FC_P) and Uganda (FC_U) (XLSX 396 kb)

Online Resource 4: Genetic correlations between pairs of environments and broad-sense heritability for dry matter (DM), starch and β-carotene (BC) measured in five environments of Peru, and flesh color (FC) measured in five environments of Peru and six environments measured in Uganda (PDF 134 kb)

Online Resource 5: Quantitative trait loci (QTL) plots for dry matter (DM), starch, β-carotene (BC), flesh color in Peru (FC_P) and Uganda (FC_U) based on a genetic map constructed from 315 progeny of a biparental mapping population between Beauregard and Tanzania (BT) sweetpotato cultivars. Black dots represent the QTL peaks, and colored bars represent their respective ~ 95% support intervals (PDF 30 kb)

Online Resource 6: Lower and Upper support intervals of the QTL peaks on the *Ipomoea trifida* and *Ipomoea triloba* reference genomes (PDF 133 kb)

Online Resource 7: Expression abundances in developing roots of Beauregard and Tanzania reported as Fragments per kb exon model per million mapped reads (FPKM). Genes underlying QTL are annotated. Expression abundances were averaged from the four biological replicates. DAT: Days after transplanting. SR: Storage roots. FR: Fibrous roots. B: Beauregard. T: Tanzania (XLSX 18929 kb)

Online Resource 8: Expression abundances (log2) in developing roots of Beauregard and Tanzania reported as Fragments per kb exon model per million mapped reads (FPKM). Expression abundances were averaged from the four biological replicates and log2 values less than zero were converted to zero. Genes underlying QTL are annotated. DAT: Days after transplanting. SR: Storage roots. FR: Fibrous roots. B: Beauregard. T: Tanzania (XLSX 6412 kb)

Online Resource 9: Pearson’s correlation coefficient analyses of replicates of Beauregard and Tanzania root development samples. Expression values (fragments per kb exon model per million mapped reads (FPKM) +1) were log2 transformed prior to analyses. Replicates are labeled a, b, c, d. DAT: Days after transplanting. SR: Storage roots. FR: Fibrous roots (PDF 49 kb)

Online Resource 10: Principal component analyses of expression profiles from Beauregard and Tanzania root development samples. Expression values (fragments per kb exon model per million mapped reads (FPKM) +1) were log2 transformed prior to analyses and represent the average of the four replicates. DAT: Days after transplanting. SR: Storage roots. FR: Fibrous roots. A. Beauregard. B. Tanzania (PDF 36 kb)

Online Resource 11: Differentially expressed genes between storage roots and fibrous roots at 30, 40, and 50 days after transplanting in Beauregard and Tanzania. DAT: Days after transplanting. SR: Storage roots. FR: Fibrous roots. B: Beauregard. T: Tanzania (XLSX 782 kb)

Online Resource 12: Biological process gene ontology enrichment in storage roots and fibrous roots in (a) Beauregard and (b) Tanzania. DAT: Days after transplanting. SR: Storage roots. FR: Fibrous roots. B: Beauregard. T: Tanzania (PDF 53 kb)

Online Resource 13: Expression abundances and differentially expressed genes (with no filtering) in developing roots of Beauregard and Tanzania reported as fragments per kb exon model per million mapped reads (FPKM) for genes involved in carotenoid metabolism. DAT: Days after transplanting. SR: Storage roots. FR: Fibrous roots. B: Beauregard. T: Tanzania. Trait abbreviations are dry matter (DM), starch, β-carotene (BC), flesh color (FC), Uganda (U), Peru (P) (XLSX 20 kb)

Online Resource 14: Differentially expressed genes between storage roots of Beauregard and Tanzania at 30, 40, and 50 days after transplanting. DAT: Days after transplanting. SR: Storage roots. B: Beauregard. T: Tanzania (XLSX 798 kb)

## Data Availability

The raw data and joint adjusted means used in QTL analyses in the current study are provided as supplementary with this manuscript (Online Resource 3). The RNA-sequencing reads are available in the NCBI Sequence Read Archive under BioProject PRJNA491292 and PRJNA549660. The phased SNPs and genetic map are available interactively at (https://gt4sp-genetic-map.shinyapps.io/bt_map/).
