# Supplementary material for: Quantitative trait loci and differential gene expression analyses reveal the genetic basis for negatively associated β-carotene and starch content in hexaploid sweetpotato [Ipomoea batatas (L.) Lam.]
Source: Theor Appl Genet. 2019 Oct 8;133(1):23–36. doi: 10.1007/s00122-019-03437-7 (PMC6952332; doi:10.1007/s00122-019-03437-7)
Supplement: Supplementary file 1 — Online Resource 1: Growing conditions for sweetpotato mapping population (BT) experiments in five environments of Peru indicating, geographic positions, elevation, mean atmospheric temperature (μTemp), mean photosynthetically active radiation (μPAR), rainfall, relative humidity, soil conditions, planting designs, planting and harvesting dates (PDF 113 kb) [file 122_2019_3437_MOESM1_ESM.pdf]

Quantitative trait loci and candidate gene expression profiles reveal the genetic basis for negatively-associated β-carotene and starch content in hexaploid sweetpotato [*Ipomoea batatas* (L.) Lam.].

Dorcus C Gemenet[1,✉,a], Guilherme da Silva Pereira[2,a], Bert De Boeck[3], Joshua C Wood[4], Marcelo Mollinari[2], Bode A Olukolu[2,11], Federico Diaz[3], Veronica Mosquera[3], Reuben T Ssali[5], Maria David[3], Mercy N Kitavi[1], Gabriela Burgos[3], Thomas Zum Felde[3], Marc Ghislain[1], Edward Carey[6], Jolien Swanckaert[6], Lachlan JM Coin[7], Zhangjun Fei[8], John P Hamilton[4], Benard Yada[9], G Craig Yencho[2], Zhao-Bang Zeng[2], Robert OM Mwanga[5], , Awais Khan[3,10], Wolfgang J Gruneberg[3], C Robin Buell[4]

[1] International Potato Center, ILRI Campus, P.O. Box 25171-00603, Nairobi, Kenya
[2] North Carolina State University, Raleigh, NC 27695, USA
[3] International Potato Center, Av. La Molina 1895, Lima, Peru
[4] Michigan State University, East Lansing, MI 48824, USA
[5] International Potato Center, Kampala, Uganda
[6] International Potato Center, Kumasi, Ghana
[7] University of Queensland, St. Lucia, Brisbane, Queensland 4072, Australia
[8] Boyce Thompson Institute, Cornell University, Ithaca, NY 14853, USA
[9] National Crops Resources Research Institute (NaCCRI), Namulonge, P.O Box 7084, Kampala, Uganda
[10] Plant Pathology and Plant-Microbe Biology Section, Cornell University, Geneva, NY, 14456, USA
[11] University of Tennessee, Knoxville, TN 37996, USA

[a] Dorcus C Gemenet and Guilherme da Silva Pereira contributed equally to this work

✉ International Potato Center, ILRI Campus, Old Naivasha Road, 25171-00603, Nairobi, Kenya; Email: d.gemenet@cgiar.org; Telephone: 254 20 422 3637; ORCID: 0000-0003-4901-1694

Online Resource 1 Growing conditions for sweetpotato mapping population (BT) experiments in five environments of Peru indicating, geographic positions, elevation, mean atmospheric temperature (µTemp), mean photosynthetically active radiation (µPAR), rainfall, relative humidity, soil conditions, planting designs, planting and harvesting dates

	**Ica16C**	**Ica16D**	**Ica17C**	**Ica17D**	**SR16**
Location	Ica				San Ramon
Latitude	14° 01' 44.7" S				11°07'29"S
Longitude	75° 44' 37.5" W				75° 21' 25" W
Altitude	420 masl				820 masl
Plot size	6 m²		4.8 m²		9 m²
µTemp	22 °C		24 °C		27 °C
µPAR	359 uE		458 uE		
Rainfall	0 mm		5 mm		122 mm

	Ica16C	Ica16D	Ica17C	Ica17D	SR16
Relative humidity	69%			66%	61%
SoilTemp	25°C	23.6°C	27 $^{\circ}$C	26 $^{\circ}$C	
Soil type	Sandy Loam				Sandy clay loam
Electrical conductivity	2.26 dS/m		0.40 dS/m		0.07 ds/m
pH	7.78		8.4		5.6
Bulk density	1.44 g/cc				
Field capacity	14.70%				
Wilting point	7.60%				
µWater potential	-233 KPa	-719 KPa	-73 KPa	-459 KPa	
Planting date	25/02/2016		15/11/2016		14/05/2016
Harvesting date	29/06/2016		17/03/2017		15/09/2016
Replications	2				3
