# Supplementary figures and images for: Quantitative trait loci and differential gene expression analyses reveal the genetic basis for negatively associated β-carotene and starch content in hexaploid sweetpotato [Ipomoea batatas (L.) Lam.]
Source: Theor Appl Genet. 2019 Oct 8;133(1):23–36. doi: 10.1007/s00122-019-03437-7 (PMC6952332; doi:10.1007/s00122-019-03437-7)

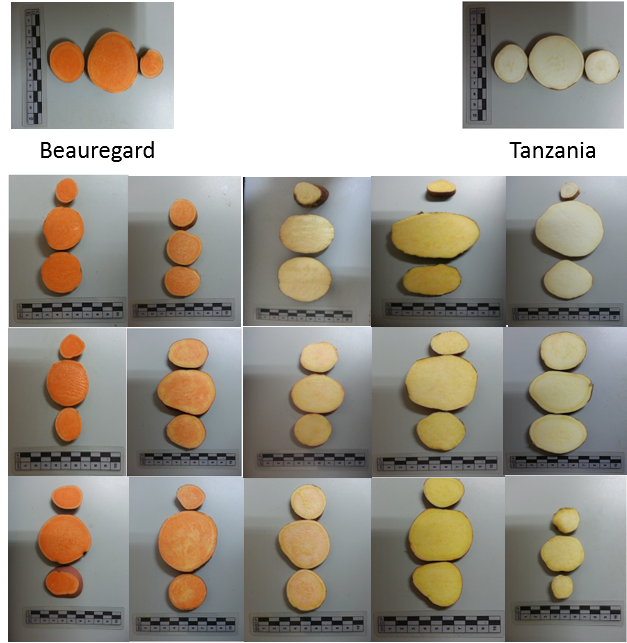

Supplement: Supplementary file 2 — Online Resource 2: Flesh color segregation in progeny as compared to parents. The progeny shown are randomly selected from the 315 population (PNG 574 kb) [file 122_2019_3437_MOESM2_ESM.png]

# Linkage Group

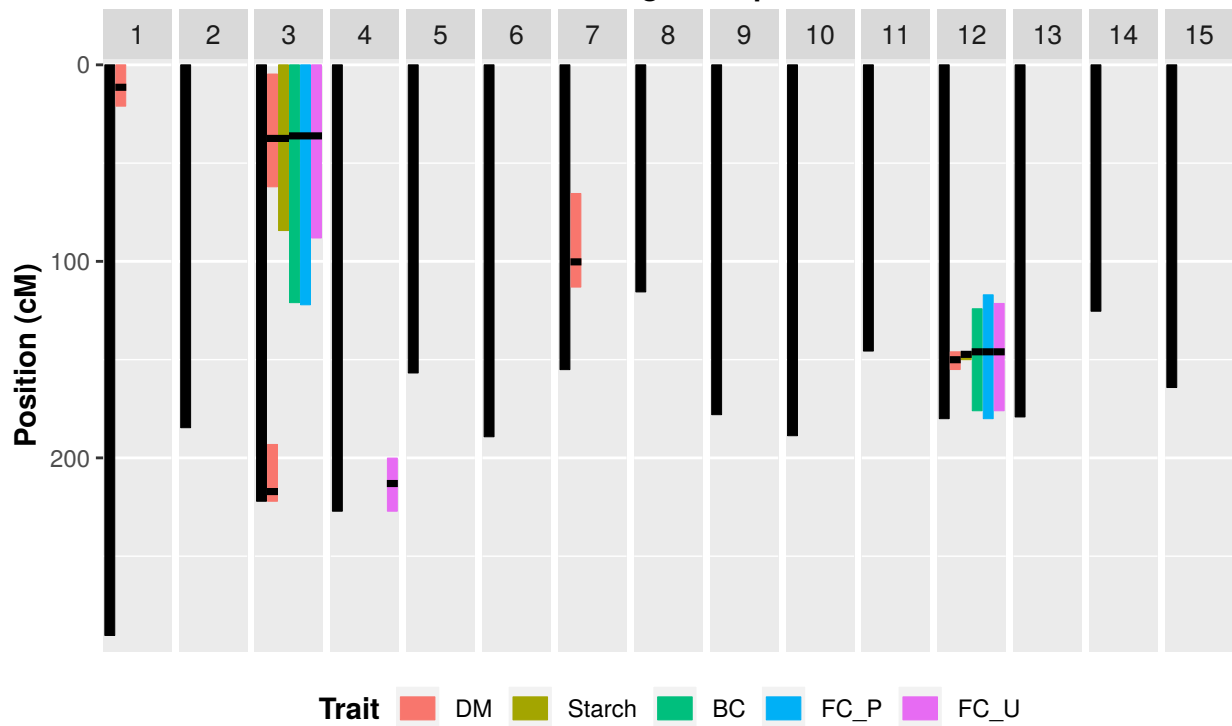

Supplement: Supplementary file 5 — Online Resource 5: Quantitative trait loci (QTL) plots for dry matter (DM), starch, β-carotene (BC), flesh color in Peru (FC_P) and Uganda (FC_U) based on a genetic map constructed from 315 progeny of a biparental mapping population between Beauregard and Tanzania (BT) sweetpotato cultivars. Black dots represent the QTL peaks, and colored bars represent their respective ~ 95% support intervals (PDF 30 kb) [file 122_2019_3437_MOESM5_ESM.pdf]

A

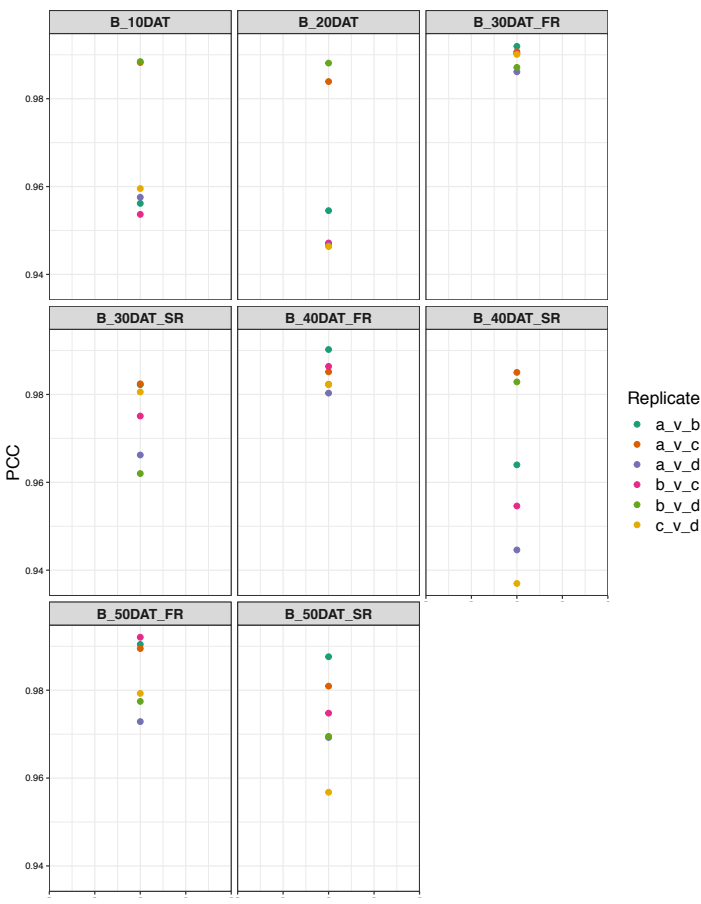

B

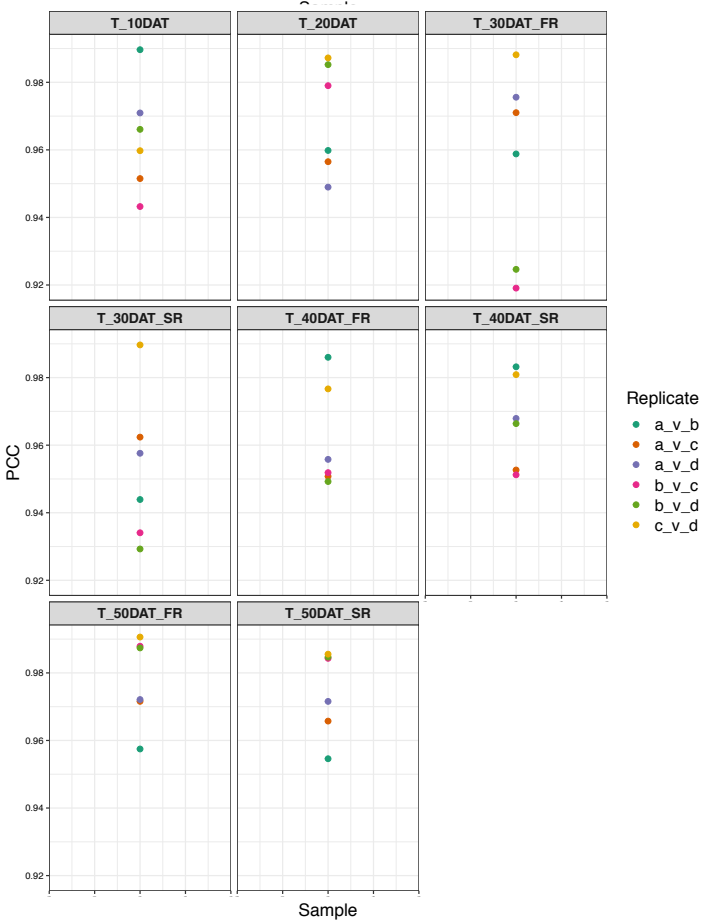

Supplement: Supplementary file 9 — Online Resource 9: Pearson’s correlation coefficient analyses of replicates of Beauregard and Tanzania root development samples. Expression values (fragments per kb exon model per million mapped reads (FPKM) +1) were log2 transformed prior to analyses. Replicates are labeled a, b, c, d. DAT: Days after transplanting. SR: Storage roots. FR: Fibrous roots (PDF 49 kb) [file 122_2019_3437_MOESM9_ESM.pdf]

A

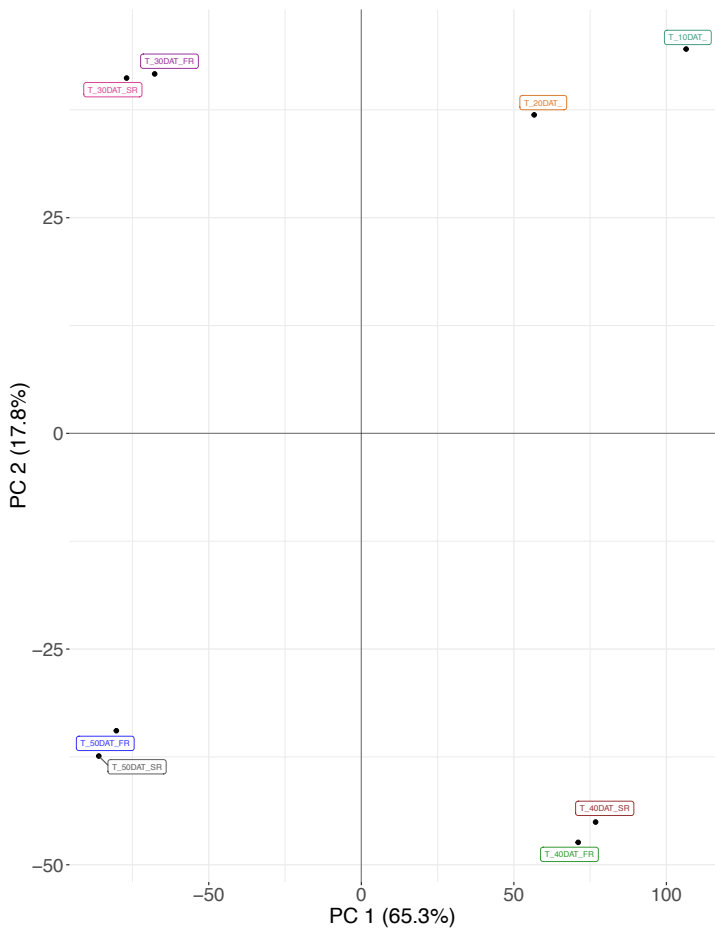

B

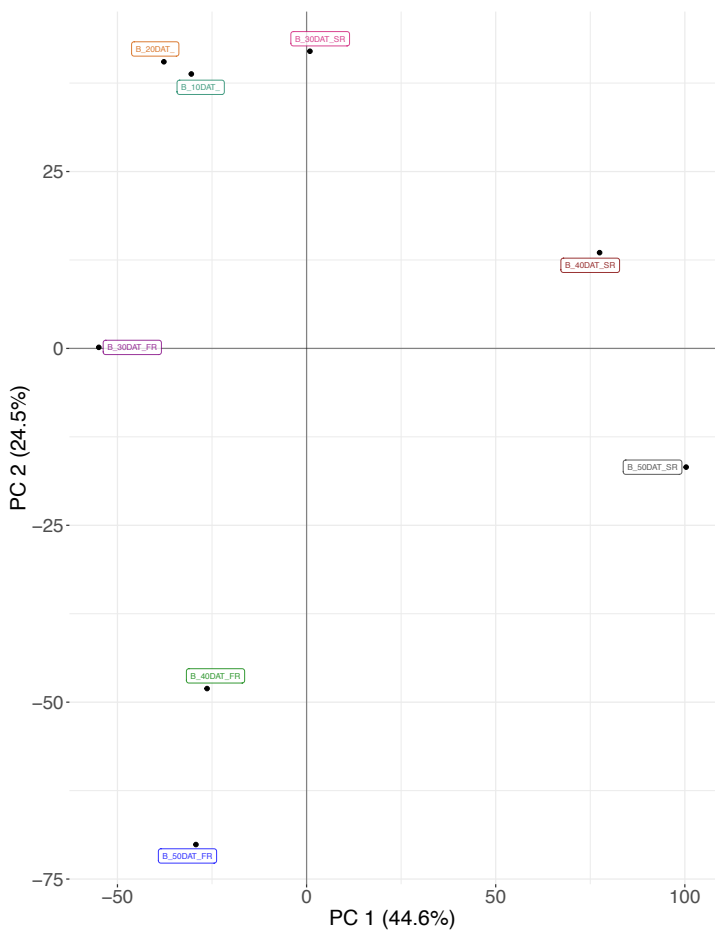

Supplement: Supplementary file 10 — Online Resource 10: Principal component analyses of expression profiles from Beauregard and Tanzania root development samples. Expression values (fragments per kb exon model per million mapped reads (FPKM) +1) were log2 transformed prior to analyses and represent the average of the four replicates. DAT: Days after transplanting. SR: Storage roots. FR: Fibrous roots. A. Beauregard. B. Tanzania (PDF 36 kb) [file 122_2019_3437_MOESM10_ESM.pdf]

Enriched BP GO Terms

### Tanzania SR vs FR Enriched BP GO Terms

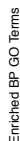

# B

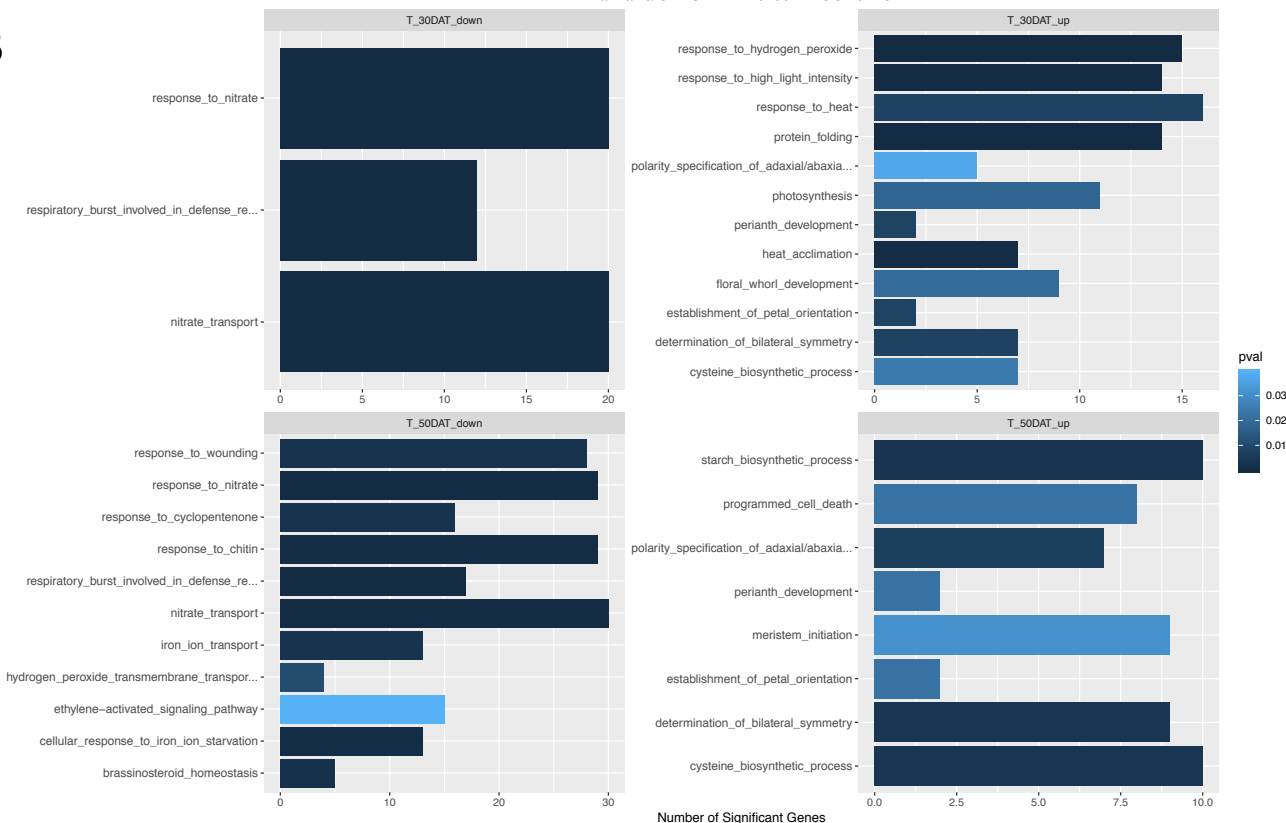

Supplement: Supplementary file 12 — Online Resource 12: Biological process gene ontology enrichment in storage roots and fibrous roots in (a) Beauregard and (b) Tanzania. DAT: Days after transplanting. SR: Storage roots. FR: Fibrous roots. B: Beauregard. T: Tanzania (PDF 53 kb) [file 122_2019_3437_MOESM12_ESM.pdf]
