# Supplementary material for: Quantitative trait loci and differential gene expression analyses reveal the genetic basis for negatively associated β-carotene and starch content in hexaploid sweetpotato [Ipomoea batatas (L.) Lam.]
Source: Theor Appl Genet. 2019 Oct 8;133(1):23–36. doi: 10.1007/s00122-019-03437-7 (PMC6952332; doi:10.1007/s00122-019-03437-7)
Supplement: Supplementary file 4 — Online Resource 4: Genetic correlations between pairs of environments and broad-sense heritability for dry matter (DM), starch and β-carotene (BC) measured in five environments of Peru, and flesh color (FC) measured in five environments of Peru and six environments measured in Uganda (PDF 134 kb) [file 122_2019_3437_MOESM4_ESM.pdf]

Quantitative trait loci and candidate gene expression profiles reveal the genetic basis for negatively-associated β-carotene and starch content in hexaploid sweetpotato [*Ipomoea batatas* (L.) Lam.].

Dorcus C Gemenet[1,✉,a], Guilherme da Silva Pereira[2,a], Bert De Boeck[3], Joshua C Wood[4], Marcelo Mollinari[2], Bode A Olukolu[2,11], Federico Diaz[3], Veronica Mosquera[3], Reuben T Ssali[5], Maria David[3], Mercy N Kitavi[1], Gabriela Burgos[3], Thomas Zum Felde[3], Marc Ghislain[1], Edward Carey[6], Jolien Swanckaert[6], Lachlan JM Coin[7], Zhangjun Fei[8], John P Hamilton[4], Benard Yada[9], G Craig Yencho[2], Zhao-Bang Zeng[2], Robert OM Mwanga[5], , Awais Khan[3,10], Wolfgang J Gruneberg[3], C Robin Buell[4]

[1] International Potato Center, ILRI Campus, P.O. Box 25171-00603, Nairobi, Kenya
[2] North Carolina State University, Raleigh, NC 27695, USA
[3] International Potato Center, Av. La Molina 1895, Lima, Peru
[4] Michigan State University, East Lansing, MI 48824, USA
[5] International Potato Center, Kampala, Uganda
[6] International Potato Center, Kumasi, Ghana
[7] University of Queensland, St. Lucia, Brisbane, Queensland 4072, Australia
[8] Boyce Thompson Institute, Cornell University, Ithaca, NY 14853, USA
[9] National Crops Resources Research Institute (NaCCRI), Namulonge, P.O Box 7084, Kampala, Uganda
[10] Plant Pathology and Plant-Microbe Biology Section, Cornell University, Geneva, NY, 14456, USA
[11] University of Tennessee, Knoxville, TN 37996, USA

[a] Dorcus C Gemenet and Guilherme da Silva Pereira contributed equally to this work

✉ International Potato Center, ILRI Campus, Old Naivasha Road, 25171-00603, Nairobi, Kenya; Email: d.gemenet@cgiar.org; Telephone: 254 20 422 3637; ORCID: 0000-0003-4901-1694

Online Resource 4 Genetic correlations between pairs of environments and broad-sense heritability for dry matter (DM), starch and β-carotene (BC) measured in five environments of Peru, and flesh color (FC) measured in five environments of Peru and six environments measured in Uganda

DM	Ica16D	Ica16C	Ica17D	Ica17C	SR16
Ica16D	0.00				
Ica16C	0.90	0.00			
Ica17D	0.86	0.91	0.00		
Ica17C	0.85	0.91	0.98	0.00	
SR16	0.82	0.87	0.94	0.92	0.00
**H2**			**0.61**		

Starch	Ica16D	Ica16C	Ica17D	Ica17C	SR16
Ica16D	0.00				
Ica16C	0.91	0.00			
Ica17D	0.93	0.96	0.00		
Ica17C	0.87	0.94	0.99	0.00	
SR16	0.86	0.90	0.96	0.94	0.00
**H2**			**0.77**		

BC	Ica16D	Ica16C	Ica17D	Ica17C	SR16
Ica16D	0.00				
Ica16C	0.94	0.00			
Ica17D	0.95	0.91	0.00		
Ica17C	0.94	0.99	0.91	0.00	
SR16	0.92	0.95	0.89	0.95	0.00
**H2**			**0.91**		

**FC**	**Peru**					**Uganda**					
	Ica16D	Ica16C	Ica17D	Ica17C	SR16	Nam16	Ser16	Kac16	Nam17	Ser17	Kac17
Ica16D	0.00										
Ica16C	0.78	0.00									
Ica17D	0.79	0.83	0.00								
Ica17C	0.78	0.81	0.88	0.00							
SR16	0.80	0.84	0.86	0.88	0.00						
Nam16	0.74	0.84	0.80	0.71	0.77	0.00					
Ser16	0.79	0.83	0.86	0.79	0.78	0.78	0.00				
Kac16	0.74	0.81	0.78	0.73	0.80	0.90	0.94	0.00			
Nam17	0.72	0.85	0.78	0.74	0.78	0.99	0.99	0.91	0.00		
Ser17	0.70	0.82	0.78	0.73	0.77	0.93	0.96	0.91	0.98	0.00	
Kac17	0.77	0.85	0.81	0.84	0.78	0.87	0.83	0.96	0.97	0.99	0.00
**H2**			**0.92**					**0.89**			
