# Supplementary material for: Quantitative trait loci and differential gene expression analyses reveal the genetic basis for negatively associated β-carotene and starch content in hexaploid sweetpotato [Ipomoea batatas (L.) Lam.]
Source: Theor Appl Genet. 2019 Oct 8;133(1):23–36. doi: 10.1007/s00122-019-03437-7 (PMC6952332; doi:10.1007/s00122-019-03437-7)
Supplement: Supplementary file 6 — Online Resource 6: Lower and Upper support intervals of the QTL peaks on the Ipomoea trifida and Ipomoea triloba reference genomes (PDF 133 kb) [file 122_2019_3437_MOESM6_ESM.pdf]

Quantitative trait loci and candidate gene expression profiles reveal the genetic basis for negatively-associated β-carotene and starch content in hexaploid sweetpotato [*Ipomoea batatas* (L.) Lam.].

Dorcus C Gemenet[1,✉,a], Guilherme da Silva Pereira[2,a], Bert De Boeck[3], Joshua C Wood[4], Marcelo Mollinari[2], Bode A Olukolu[2,11], Federico Diaz[3], Veronica Mosquera[3], Reuben T Ssali[5], Maria David[3], Mercy N Kitavi[1], Gabriela Burgos[3], Thomas Zum Felde[3], Marc Ghislain[1], Edward Carey[6], Jolien Swanckaert[6], Lachlan JM Coin[7], Zhangjun Fei[8], John P Hamilton[4], Benard Yada[9], G Craig Yencho[2], Zhao-Bang Zeng[2], Robert OM Mwanga[5], , Awais Khan[3,10], Wolfgang J Gruneberg[3], C Robin Buell[4]

[1] International Potato Center, ILRI Campus, P.O. Box 25171-00603, Nairobi, Kenya
[2] North Carolina State University, Raleigh, NC 27695, USA
[3] International Potato Center, Av. La Molina 1895, Lima, Peru
[4] Michigan State University, East Lansing, MI 48824, USA
[5] International Potato Center, Kampala, Uganda
[6] International Potato Center, Kumasi, Ghana
[7] University of Queensland, St. Lucia, Brisbane, Queensland 4072, Australia
[8] Boyce Thompson Institute, Cornell University, Ithaca, NY 14853, USA
[9] National Crops Resources Research Institute (NaCCRI), Namulonge, P.O Box 7084, Kampala, Uganda
[10] Plant Pathology and Plant-Microbe Biology Section, Cornell University, Geneva, NY, 14456, USA
[11] University of Tennessee, Knoxville, TN 37996, USA

[a] Dorcus C Gemenet and Guilherme da Silva Pereira contributed equally to this work

✉ International Potato Center, ILRI Campus, Old Naivasha Road, 25171-00603, Nairobi, Kenya; Email: d.gemenet@cgiar.org; Telephone: 254 20 422 3637; ORCID: 0000-0003-4901-1694

Online Resource 6. Lower and Upper support intervals of the QTL peaks on the *Ipomoea trifida* and *Ipomoea triloba* reference genomes

			Peak (cM)			Lower SI			Upper SI		
Trait	QTL	LG	Pos	I. trifida	I. triloba	Pos	I. trifida	I. triloba	Pos	I. trifida	I. triloba
DM	1	1	11.41	1219666	1403965	0.00	29275	1326265	21.02	1617558	1960333
	2	3	37.44	3185578	3524655	4.68	161515	167255	62.06	5077317	5798830
	3	3	217.13	11501067	13981098	193.17	11050542	13451516	222.06	12722755	15276175
	4	7	100.26	13656957	9559232	65.46	4871286	5298086	113.08	20729361	16191460
	5	12	150.05	22369268	26377736	146.02	22131994	26093277	155.04	22677238	26760596
Starch	1	3	37.44	3185578	3524655	0.00	32030	22131	84.32	8376264	25401733
	2	12	147.31	22197168	26179595	146.02	22131994	26093277	150.05	22369268	26377736
BC	1	3	36.14	2994719	3301261	0.00	32030	22131	121.00	18685468	29618516
	2	12	146.02	22131994	26093277	124.12	20619123	24237722	176.02	23814088	27965895
FC_P	1	3	36.14	2994719	3301261	0.00	32030	22131	122.10	18578085	29500157
	2	12	146.02	22131994	26093277	117.02	20029940	23619227	180.05	24094782	28283707
FC_U	1	3	36.14	2994719	3301261	0.00	32030	22131	88.10	24546064	26538223
	2	4	213.05	31363290	35012593	200.09	30146741	33756387	227.13	32464111	36265727
	3	12	146.02	22131994	26093277	121.41	20288552	23928750	176.02	23814088	27965895
